# Cultural Context or Generational Cohort: Which Influences Tourist Behavior More?

**DOI:** 10.3389/fpsyg.2022.767035

**Published:** 2022-02-17

**Authors:** Gema Pérez-Tapia, Pere Mercadé-Melé, Hwang Yeong-Hyeon, Fernando Almeida-García

**Affiliations:** ^1^Department of Economy and Business Administration, University of Malaga, Málaga, Spain; ^2^Department of Statistics and Econometrics, University of Malaga, Málaga, Spain; ^3^Department of Tourism Management, Dong-A University, Busan, South Korea; ^4^Department of Geography, University of Malaga, Málaga, Spain

**Keywords:** millennials, personality traits, generational culture, travel personality, travel motivations

## Abstract

According to most academics, different generations share common characteristics. This undoubtedly helps to better understand their behavior in different scenarios, predicting their responses. However, this seems questionable and that is the main purpose of this study. This research, although preliminary, try to confirm if millennials have common characteristics, or if, on the contrary, there are differences between them due to the culture in which they are immersed. To this end, it has been contextualized in a sector that is very sensitive to cultural differences, such as tourism. Data collection was carried out through a questionnaire administered in 2019 *via* e-mail to young South Korean and Spanish millennials (born 1982–2002). The results suggest that there do not appear to be common personality traits between the countries analyzed. The differences between the two groups for most personality traits and travel motivations were significant. The findings of this research provide several practical implications since it will make possible a more appropriate management of the tourist destination.

## Introduction

In a globalized world, where uncertainty caused by the COVID-19 pandemic and competition in markets predominate, it becomes imperative to know the behavior of our current and potential consumers, to have the ability to succeed in decisions. In the tourism sector, this is happening in a dizzying way, and for companies and tourist institutions to know the behavior of tourist and the factors that affect them is much needed. This is especially true in regard to millennials as Parsa and Cobanoglu (2011) claim this generation is becoming an important source of tourists in some destinations.

In general, generational cohorts derived from shared values, preferences, and beliefs can provide stable results in terms of their preferred travel behavior and consumption patterns (Schewe and Meredith, 2004; Benckendorff et al., 2010).

Millennials are characterized by being more materialistic than members of previous groups, and of giving more importance to their image (Twenge, 2014). They are also characterized by being multitasking, have important digital skills, and in general, have a high level of education, are energetic, positive, socially responsible, sophisticated, and demanding (Ng et al., 2010; Pendergast, 2010).

Compared to previous generations, millennials demonstrate more self-confidence (Myers and Sadaghiani, 2010) and higher self-esteem (Logan, 2008).

Also, this group is characterized by strong consumption of audiovisual products, important participation in and use of social networks, and great dependence on the internet. In addition, this group is more open to different cultures and languages, and to traveling internationally from an early age (Ahn et al., 2020). Social media has changed the lives of millennials, allowing them to express their personalities through social networks. This allows you to get to know and be imbued with other cultures.

For the tourism industry, being able to predict behavior based on generational cohorts is a great advantage because depending on a date of birth it can foresee patterns of behavior, needs and unsatisfied desires as well as tourist motivations that help the definition and design of the tourism product as well as its communication. This premise (based on the homogeneity of tourists according to their generational cohort) facilitates the management and ensures the success of any tourist destination.

But this generalization is certainly questionable. Some research is already appearing that questions this fact and shows that tourists born in a period of time are not as similar as one might expect. Two recent publications stand out in this regard. On the one hand, the research carried out by Bachman et al. (2021) in which, after analyzing two groups of millennials who choose and visit two types of tourist attractions, it is concluded that despite the socio-cultural similarities, there are significant differences between both groups of millennials in terms of social involvement, self-image congruency, and social return. And on the other hand, the study proposed by Garikapati et al. (2016) in which a longitudinal analysis is carried out and several groups within the generational cohort are distinguished. Within the millennials analyzed, it is shown that their behavior and needs change over time. Young millennials have characteristics that change as time goes on.

This leads us to think that bringing together all those born in a long period of time is not appropriate, since they change their behaviors as they turn years old. If there are differences between the subjects themselves who belong to a particular generational cohort, one might think that they cannot all be treated equally. If we add to this the obvious cultural differences that authors such as Hofstede (2011) have studied, this is even more relevant.

From this reflection, it would be appropriate to look for another factor of influence on behavior that helps predict behaviors and that is not so fickle and that remains over time. In this way, it would facilitate the understanding of their characteristics and behaviors, making it easier for the tourism industry to manage destinations and contributing to their growth.

The following question then arises: Could culture be that variable or factor of influence that really defines tourist motivations and therefore behavior? This reflection is supported by the numerous investigations that suggest that culture is an important factor of influence on the behavior of tourists (Meng, 2010).

In this research, culture will be measured from the personality, having as a premise what some academics defend, which is the assimilation of both concepts. Traditionally, concepts of culture and personality have been considered distinct concepts. However, there are authors who assimilate personality and culture as very close elements, considering culture as something shared by members of a society or nation and personality being an individual trait.

The concept of personality does not have a universal meaning. There are researches that describe it as a dynamic system that generates characteristic patterns such as the behavior, thoughts, and feelings of the individual (Allport, 1961), while others associate it with individuals’ relatively enduring patterns of cognition, emotion, and behavior that distinguish one person from another (Roberts et al., 2008).

On the other hand, national culture can drive or guide consumer attitudes and their subsequent behavior (Craig and Douglas, 2006). This culture includes elements such as beliefs, shared values, and norms that distinguish it from another group or culture (Pizam et al., 1997; Hofstede, 2001). In the tourism sector, the role of national culture in shaping patterns of tourist behavior has long been recognized (Kang and Moscardo, 2006).

In fact, after the introduction of the concept of “individual cultural values” by Yoo et al. (2011), both concepts (culture and personality) have been equated in a sense and have been regarded as influencers on individual behavior. The literature provides several studies in which both concepts are related and treated as similar concepts (Costa et al., 2001; Hofstede and McCrae, 2004).

From this perspective in which culture and personality converge, it is considered necessary to deepen the differences between nations/cultures. Culture represents a key dimension in tourism, and its interest is growing among researches. This interest is reflected in the growing number of studies that are including culture as a crucial variable in the research carried out (Master and Prideaux, 2000; Crotts and Pizam, 2003; Cohen and Cohen, 2012; He and Filimonau, 2020).

Nowadays, however, the world is characterized by having “liquid borders,” which would explain a tendency of homogenization and of following similar patterns of conduct (personality) whatever the country of origin/residence. Above all, if we look at the new generations, it seems that they are becoming more and more similar and that cultural differences are no longer key in behavior, giving way to hyperconnected generations that are imbued with other cultures and that behave in a similar way.

The main objective of this work is to analyze whether, as most academics defend, the generational cohort is a factor of influence on tourism behavior, defining common patterns and similar responses or whether, on the contrary, it is the culture, measured in terms of personality traits, that effectively underlies and marks the behavior of its tourists.

Analyzing this dichotomy is the main novelty of this research work, which will focus on millennials as a group of consumers, as they represent an important economic force very influential in new trends in consumption and are present and future consumers.

The results of this preliminary study might suggest two different scenarios. On the one hand, that generational cohorts are effectively prescribers of tourism behavior and that they should be considered as homogeneous groups. On the other hand, that culture is the one that mainly defines the behavior and not the date of birth. Both scenarios would have important implications at the level of design, management, and communication of tourist destinations.

## Methodology

The research was conducted based on a structured questionnaire that was administered between April and October 2019 *via* e-mail to young South Korean and Spanish millennials (born between 1982 and 2002). The reason for the choice of these two nationalities is that *a priori* they are two quite contraposed cultures (House et al., 2004), but on the other hand they are two remarkably similar countries in terms of level of economic growth and development, number of inhabitants and technological development, which could imply a “normalization or homogenization” of their young people that might result in common patterns of behavior.

The convenience sampling method was used, because the subject being analyzed required a specific population segment: Korean and Spanish millennials. It is important to note, that, although the sampling method was not probabilistic, convenience sampling is often used in social research. There are authors who defend this sampling method (Sirakaya et al., 2003) when it is difficult to obtain a complete sampling frame. This practice is also common in the tourism field due to the difficulty involved in obtaining large samples of the study population (Bigné et al., 2012).

Korean and Spanish millennials, the nationalities under study in this research, also share similarities in relation to internet and social media consumption (Table 1).

**TABLE 1:** Internet and social media consumption in South Korea and Spain (April 2020).

	Mobile connections	Internet users	Active social media users
South Korea (51.29 million of population)	60.67 million (118.3% of population)	49.75 million (97% of population)	45.79 million (89.3% of population)
Spain (46.75 million of population)	54.34 million (116.2% of population)	42.54 million (91% of population)	37.40 million (80% of population)

*Source: https://wearesocial.com/blog/2020/04/digital-around-the-world-in-april-2020/.*

To facilitate their understanding, the questionnaire was translated into both Spanish and Korean, thus avoiding errors of interpretation. The questionnaire consists of three distinct parts. The first part aims to measure personality traits and travel personality. The second part includes the motivations of the Korean and/or Spanish tourist when choosing a destination. The last section covers issues relating to sociodemographic aspects, among others, gender, and level of studies.

To measure the main variables, the scales used are based on a wide review of the literature and on scales proposed by academics of proven relevance (supporting their reliability and validity).

After the collection and debugging of the information, 286 of a total of 304 surveys have been valid, the sampling error would be 5.8% with a 95% confidence level in the worst case of *p* = *q* = 0.5, being adequate in the field of experimental behavioral research (Abranovic, 1997; Hill, 1998). In addition, to check the sufficiency of the data, split-half analysis of consistency by Martin and Bateson (1986) has been carried out, where the data have been randomly divided into two halves and analyzed separately and the same conclusions have been obtained. Table 2 presents the characteristics of the sample.

**TABLE 2:** Sociodemographic variables (N 304).

	Variables	Frequency	%
Nationality	Korean	159	55.6
	Spanish	127	44.4
Gender	Male	76	26.6
	Female	210	73.4
Studies	Primary/secondary	203	71.0
	University/postgraduate	83	29.0
			

There are several approaches to measuring personality. In this work we use that proposed by Gosling et al. (2003), which is the Ten-Item Personality Inventory (TIPI) from the Big Five. The study conducted by Gosling et al. (2003) proposes several methods to reduce the scale of personality measurement and concludes that the Ten-Personality Item Inventory (TIPI) has adequate levels of (i) convergence with the widely used Big Five scale, and (ii) reliability and patterns of predicted external correlates. The respondent is therefore asked about ten variables that are measured from a Likert scale of 7 points ranging from 1 (strongly disagree) to 7 (strongly agree).

The scale proposed by Gretzel et al. (2004) has been used to measure travel personality. This scale consists of 12 travel personalities, in which the respondent must sort 12 according to the one that best and worst describes it.

Finally, motivation was measured through the scale proposed by Beerli and Martin (2004), who established 14 travel motivations. Respondents valued on a 7-point Likert scale (1: nothing important; 7: very important) the importance of each of the proposed reasons when visiting a destination.

## Findings

After a first approximation of the information obtained through the questionnaires, some relevant results are highlighted. Table 3 shows the personality traits measured through the scale TIPI from the Big Five.

**TABLE 3:** Personality traits.

Traits of Personality: TIPI	Nationality			
	Korean	Spanish	*t*-student difference in means	*p*-value
Extraverted, enthusiastic	4.94 ± 1,525	5.02*sw*±1.561	–0.472	0.637
Critical, quarrelsome	4.13 ± 1.684	3.35 ± 1.738	3.864	0.000***
Dependable, self-disciplined	5.4 ± 0.948	5.44 ± 1.295	–0.326	0.745
Anxious, easily upset	4.30 ± 1.569	3.54 ± 1.722	3.858	0.000***
Open to new experiences	5.70 ± 1.156	6.04 ± 1.171	–2.421	0.016**
Reserved, quiet	3.87 ± 1.683	4.16 ± 1.941	–1.30	0.1985
Sympathetic, warm	5.47 ± 1.135	6.00 ± 1.106	–3.968	0.000***
Disorganized, careless	3.86 ± 1.686	3.72 ± 1.934	0.631	0.529
Calm, emotionally stable	4.22 ± 1.483	4.63 ± 1.689	–2.151	0.032**
Conventional, uncreative	3.84 ± 1.350	3.03 ± 1.623	4.579	0.000***

**p-value < 0.05; and ***p-value < 0.01. Statiscal analysis: Mean ± standard deviation, T-student, and p-value.

**FIGURE 1:**
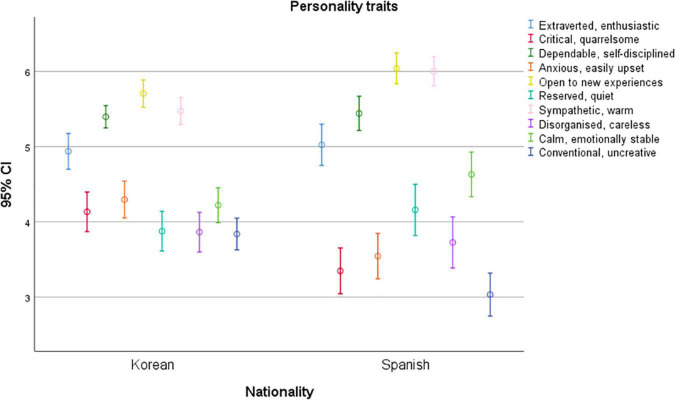
Personality traits by nationality.

There are some significant differences between the personalities of Korean and Spanish millennials (Figure 1). This result would confirm the assumptions and hypotheses on which this research is based: the characteristics and behaviors (personality) of millennials cannot be generalized, since there are significant differences between them. In such a way, it supports the conclusions proposed by Garikapati et al. (2016) and Bachman et al. (2021) in their previous research.

Figure 1 shows the averages for the personality assessment and calculates contrasts for mean differences in two independent populations, Korean and Spanish. There are significant differences in being critical (*t* = −3.864; *p* = 0.000), anxious (*t* = −3.858; *p* = 0.000), open to new experiences (*t* = −2.421; *p* = 0.016), sympathetic (*t* = −3.968; *p* = 0.000), calm and emotionally stable (*t* = −2.151; *p* = 0.032), and conventional and uncreative (*t* = −4.579; *p* = 0.000). Statistically significant differences were not seen for other personality traits.

According to this, Koreans seem more prone to critical thinking and are easily angered, more nervous, more upset, and more traditional and uncreative than Spaniards. In contrast, Spaniards are found to be more open to new experiences, understanding, affectionate, calm, and emotionally stable than Koreans. In the other personality traits, extroverted and enthusiastic, reliable and self-disciplined, reserved and quiet, and disorganized and careless, no statistically significant differences can be seen.

The results for travel personality are shown in Table 4.

**TABLE 4:** Frequency distribution of travel personality categories.

	That describes best		That describes least	
Travel Personality	Korean	Spanish	Korean	Spanish
Culture creature	27.7%	17.3%	0.0%	3.1%
City slicker	3.1%	16.5%	1.3%	3.9%
Sight seeker	16.4%	14.2%	0.0%	0.0%
Family guy	8.8%	14.2%	2.5%	1.6%
Beach boom	9.4%	11.0%	1.3%	3.9%
Avid athlete	1.3%	2.4%	20.8%	7.9%
Shopping shark	8.8%	6.3%	5.0%	5.5%
All arounder	13.8%	6.3%	0.6%	4.7%
Trail trekker	7.5%	3.9%	3.1%	2.4%
History buff	1.3%	6.3%	0.0%	8.7%
Boater	0.6%	0.0%	14.5%	8.7%
Gamer	1.3%	1.6%	34.6%	49.6%

The personality that best defines Koreans is the “Cultural Creature,” with 27.7% of respondents, followed by the “Landscape Seeker” (16.4%). Among Spanish millennials, the first option that best defines their personality is the “Cultural Creature” (17.3%), followed by “City Slicker” (16.5%). For both groups, the personality trait that defines them least is “Gamer.”

As for the “Travel personality” if it could be said that there are some similarities, as suggested by authors such as (Schewe and Meredith, 2004; Benckendorff et al., 2010). It could also be interpreted to coincide with what Ahn et al. (2020) suggest in the sense that millennials share an interest in knowing different cultures.

Finally, and in terms of tourist motivations for Koreans and Spaniards (Table 5), there are statistically significant differences observed in all tourist motivations except “Escaping the daily routine” and “Living exciting experiences,” where there were no nationality-based differences. Koreans attached more importance to “rest/relax” (*t* = −3.768; *p* = 0.000) and “release stress and tension” (*t* = −3.632; *p* = 0.000). In contrast, the Spanish gave more importance to the desire to “know new places” (*t* = −8.671; *p* = 0.000), “know the natural environment” (*t* = −4.913; *p* = 0.000), “know historical heritage” (*t* = −7.77; *p* = 0.000), “know the culture and way of life” (*t* = −7.168; *p* = 0.000), “meet new people” (*t* = −3.323; *p* = 0.001), “integrate into the lives and activities of local people” (*t* = −3.107; *p* = 0.002), “enjoy with family and/or friends” (*t* = −4.066; *p* = 0.000), “seek adventures” (*t* = −6.75; *p* = 0.000); “get close to nature” (*t* = −6.977; *p* = 0.000), and “seek entertainment and fun” (*t* = −6.977; *p* = 0.000).

**TABLE 5:** Tourist motivations.

Tourist motivations	Nationality			
	Korean	Spanish	*t-*student	*p-value*
Rest/relax	5.67 ± 1.32	4.97 ± 1.723	3.768	0.000***
Relieve stress and tension	6.14 ± 1.034	5.58 ± 1.455	3.632	0.000***
Escaping the daily routine	6.51 ± 0.826	6.48 ± 0.967	0.275	0.784
Meet new places	5.62 ± 1.256	6.69 ± 0.804	8.671	0.000***
Know your natural environment	4.63 ± 1.324	5.45 ± 1.494	–4.913	0.000***
Know its historical heritage	3.77 ± 1.542	5.19 ± 1.516	–7.770	0.000***
Know your culture and way of life	4.62 ± 1.475	5.80 ± 1.279	–7.168	0.000***
Meet new people	4.33 ± 1.633	4.99 ± 1.707	–3.323	0.001***
Integrating into the lives and activities of local people.	4.09 ± 1.632	4.69 ± 1.640	–3.107	0.002***
Enjoy with my family and/or friends	5.96 ± 1.174	6.48 ± 0.925	–4.066	0.000***
Search for adventures	4.55 ± 1.679	5.76 ± 1.354	–6.750	0.000***
Being in touch with nature	4.09 ± 1.534	4.80 ± 1.574	–3.839	0.000***
Search for entertainment and fun	5.25 ± 1.382	6.21 ± 0.94	–6.977	0.000***
Live exciting experiences	6.22 ± 1.023	6.38 ± 1.007	–1.305	0.193

***p-value < 0.01. Statiscal analysis: Mean ± standard deviation, T-student, and p-value.

## Conclusion

The results suggest that, although millennials have common characteristics such as being technologically intelligent (Maxwell et al., 2010), being open to different cultures (Schewe and Meredith, 2004; Benckendorff et al., 2010; Meng, 2010), and frequently traveling (Ahn et al., 2020), it should be noted that in terms of personality they do not present common patterns or traits.

It seems that culture, measured in terms of personality traits, prevails, and resists the lure of globalization. The differences between the two groups are significant. These differences identified in this investigation could influence travel motivations and therefore tourists’ behavior, being in line with what other authors claim (Kozak, 2002; Lee and Lee, 2009; Hofstede, 2011; Whang et al., 2016; Bachman et al., 2021).

Indeed, the results of this research also suggest that tourist motivations differ between the two groups. Korean citizens attach more importance to relaxation and travel mainly to rest and relieve stress, while Spanish citizens consider knowing new places, the natural environment, and historical heritage to be the main reason they travel. This could be explained by their previously identified personality traits. Koreans are more nervous or feel more anxious, and this could explain the search for relaxation on their travels. On the other hand, Spaniards stand out for being open to new experiences, which could be related to the need to know new places, their natural environment, their historical heritage, and their culture and ways of life.

However, as for the travel personality that best describes both groups, the differences are not so noticeable, with culture and cultural representations being the main attraction for both.

From these preliminary results it is intended to determine whether there is an influencing relationship between personality traits and travel personality, as well as whether personality traits influence tourist motivations. In tourism, personality is a determining factor in tourist motivations, perception, and behavior (Swarbrooke and Horner, 2012).

This study provides conceptual and theoretical progress and implications for tourism research. Conceptually, this research delves into one of the most influential factors in behavior. Personality is constituted as a key element in the motivations in the process of choosing the tourist destination.

This research establishes a very close relationship between personality and behavior, leaving in the background the generational cohort as an explanatory factor of behavior. Therefore, this preliminary research provides further evidence and support the results of academics as Garikapati et al. (2016) and Bachman et al. (2021).

*A priori*, it could be said that personality traits define consumers more and better than the generation to which they belong. In order to generalize this statement, a longitudinal analysis would need to be done over time to see if these differences are maintained or instead softened.

All this, consequently, will have important implications in terms of the management of the tourist destinations themselves. Identifying motivations and preferred activities depending on the cultural environment in which potential tourists develop is an element of influence, not being so relevant the age of them. Being able to relate a type of culture to certain tourist motivations has important implications for DMOs. The tourist destination could be adapted to offer the most appropriate activities to each group.

This research presents as its main limitation the sample, since the study is only carried out for two specific cultures or nationalities (Korean and Spanish) and is also done in a transversal way. Future lines of research should conduct this same study in different countries and using some model that also makes it possible to identify factors or variables of influence and longitudinal analysis (Garikapati et al., 2016). Also, it could be interesting to analyze the possible effect of the pandemic caused by COVID-19 in this aspect by performing a temporal analysis (and comparing both results).

## Data Availability Statement

The raw data supporting the conclusions of this article will be made available by the authors, without undue reservation.

## Author Contributions

GP-T, FA-G, and HY-H contributed to the conception and design of the study. PM-M organized the database and performed the statistical analysis. GP-T wrote the first draft of the manuscript. All authors contributed to manuscript revision, read, and approved the submitted version.

## Conflict of Interest

The authors declare that the research was conducted in the absence of any commercial or financial relationships that could be construed as a potential conflict of interest.

## Publisher’s Note

All claims expressed in this article are solely those of the authors and do not necessarily represent those of their affiliated organizations, or those of the publisher, the editors and the reviewers. Any product that may be evaluated in this article, or claim that may be made by its manufacturer, is not guaranteed or endorsed by the publisher.
